# PEDF-derived peptide promotes tendon regeneration through its mitogenic effect on tendon stem/progenitor cells

**DOI:** 10.1186/s13287-018-1110-z

**Published:** 2019-01-03

**Authors:** Tsung-Chuan Ho, Shawn H. Tsai, Shu-I Yeh, Show-Li Chen, Kwang-Yi Tung, Hsin-Yu Chien, Yung-Chang Lu, Chang-Hung Huang, Yeou-Ping Tsao

**Affiliations:** 1 Department of Medical Research, Mackay Memorial Hospital, No. 45, Minsheng Rd., Tamsui District, New Taipei City, 25160 Taiwan; 2 Department of Ophthalmology, Mackay Memorial Hospital, No. 92, Sec. 2, Chung Shan North Road, Taipei, 10449 Taiwan; 3 Department of Optometry, Chung Shan Medical University, Taichung, 40201 Taiwan; 4 Department of Microbiology, School of Medicine, National Taiwan University, No. 1 Jen Ai road, section 1, Taipei, 100 Taiwan; 5 Department of Plastic Surgery, Mackay Memorial Hospital, No. 92, Sec. 2, Zhongshan N. Rd., Taipei, 10449 Taiwan; 6 Departments of Biomechanics Laboratory, and Orthopaedic Surgery, Mackay Memorial Hospital, No. 45, Minsheng Rd., Tamsui District, New Taipei City, 25160 Taiwan; 7 Department of Dentistry, National Yang-Ming University, Taipei, Taiwan

**Keywords:** PEDF, Peptide, Tendon stem/progenitor cell, Signaling

## Abstract

**Background:**

Tendon stem/progenitor cells (TSPC) exhibit a low proliferative response to heal tendon injury, leading to limited regeneration outcomes. Exogenous growth factors that activate TSPC proliferation have emerged as a promising approach for treatment. Here, we evaluated the pigment epithelial-derived factor (PEDF)-derived short peptide (PSP; 29-mer) for treating acute tendon injury and to determine the timing and anatomical features of CD146- and necleostemin-positive TSPC in the tendon healing process.

**Methods:**

Tendon cells were isolated from rabbit Achilles tendons, stimulated by the 29-mer and analyzed for colony-forming capacity. The expression of the TSPC markers CD146, Oct4, and nestin, induced by the 29-mer, was examined by immunostaining and western blotting. Tendo-Achilles injury was induced in rats by full-thickness insertion of an 18-G needle and immediately treated topically with an alginate gel, loaded with 29-mer. The distribution of TSPC in the injured tendon and their proliferation were monitored using immunohistochemistry with antibodies to CD146 and nucleostemin and by BrdU labeling.

**Results:**

TSPC markers were enriched among the primary tendon cells when stimulated by the 29-mer. The 29-mer also induced the clonogenicity of CD146^+^ TSPC, implying TSPC stemness was retained during TSPC expansion in culture. Correspondingly, the expanded TSPC differentiated readily into tenocyte-like cells after removal of the 29-mer from culture. 29-mer/alginate gel treatment caused extensive expansion of CD146^+^ TSPC in their niche on postoperative day 2, followed by infiltration of CD146^+^/BrdU^−^ TSPC into the injured tendon on day 7. The nucleostemin^+^ TSPC were located predominantly in the healing region of the injured tendon in the later phase (day 7) and exhibited proliferative capacity. By 3 weeks, 29-mer-treated tendons showed more organized collagen fiber regeneration and higher tensile strength than control tendons. In culture, the mitogenic effect of the 29-mer was found to be mediated by the phosphorylation of ERK2 and STAT3 in nucleostemin^+^ TSPC.

**Conclusions:**

The anatomical analysis of TSPC populations in the wound healing process supports the hypothesis that substantial expansion of resident TSPC by exogenous growth factor is beneficial for tendon healing. The study suggests that synthetic 29-mer peptide may be an innovative therapy for acute tendon rupture.

## Background

Tendons contain dense connective tissues and mediate the transmission of muscle force to the bone, which is crucial for the control of body movement. Tendon injuries are common and often caused by overstretching the tendon. However, tendons have limited ability for self-healing following severe injury, because of their avascularity and acellularity [1, 2]. Unlike another type of connective tissue, bone marrow mesenchymal stromal cells (BM-MSCs) are difficult to mobilize into the injured sites of tendons [1]. The repair of tendons is therefore a slow and relatively difficult process. Recently, intensive efforts have been made to use cell therapy-based approaches to accelerate tendon regeneration and repair [1]. Adult mesenchymal stromal cells (MSCs) can be obtained to provide an adequate cellular source for tendon regeneration [1]. However, cell transplantation is a time-consuming process. In addition, cost and technical and safety issues are obstacles to providing benefits to patients [3, 4]. Growth factors injected into and around the injured site to stimulate tendon stem/progenitor cells (TSPC) proliferation may offer an alternative option for promoting tendon repair. Connective tissue growth factor (CTGF) can promote tendon wound healing by stimulating proliferation of a TSPC population marked by CD146. The CD146^+^ TSPC cluster the tendon periphery, mainly near the blood vessels [3]. Platelet-rich plasma (PRP) that harbors platelet-derived growth factor (PDGF) is another means of treating tendon injury, although the effect is limited, possibly because of a low concentration of PDGF in the preparation [5]. It has been reported that hydrogel combinations of fibroblast growth factor (FGF)-2, insulin-like growth factor (IGF)-1, and PDGF-BB can improve the survival of adipose-derived mesenchymal stem cells (ASCs) and aid tendon healing [6, 7]. The advantage of growth factor treatment is that it is readily available for acute tendon injury, avoiding the waiting period of cell therapy. In addition, several growth factors have the ability to induce TSPC proliferation in culture. CTGF can enhance the clonogenic capacity of CD146^+^ TSPC [3]. FGF-2 promotes the growth of TSPC marked by expression of scleraxis (Scx) and SRY-box containing gene 9 (Sox9) [8]. However, the mitogenic signaling stimulating TSPC proliferation remains largely unknown.

Pigment epithelial-derived factor (PEDF) has been reported to mediate the proliferation of several stem/progenitor cell populations. PEDF is effective in stimulating the proliferation of neuronal progenitor cells and human embryonic stem cells [9, 10]. Recent studies demonstrated further that PEDF and pigment epithelial-derived factor-derived short peptide (PSP) (29-mer; residues Ser93-Thr121) can stimulate the proliferation of limbal stem cells, muscle satellite cells, and hepatic stem cells [11–13]. Moreover, PSP has been suggested to have a potential for treating several types of tissue injury [11–13]. In this study, we investigated the potential application of the 29-mer to the healing of Achilles tendon ruptures in animals. The mitogenic activity of the 29-mer on TSPC in vitro was also explored.

## Methods

### Materials

The PSP 29-mer (Ser93-Thr121; SLGAEQRTESIIHRALYYDLISSPDIHGT) and 34-mer (Asp44-Asn77; DPFFKVPVNKLAAAVSNFGYDLYRVRSSTSPTTN) were synthesized, modified by acetylation at the NH_2_ termini and amidation at the COOH termini for stability, and characterized by mass spectrometry (> 90% purity) at GenScript (Piscataway, NJ). Dulbecco’s modified Eagle’s medium (DMEM), fetal bovine serum (FBS), antibiotic–antimicotic solutions, and trypsin were purchased from Invitrogen (Carlsbad, CA). 5-Bromo-2′-deoxyuridine (BrdU), insulin–transferrin–sodium selenite (ITSE) media supplement, Hoechst 33258 dye, Trichrome Stain (Masson) Kit, and all chemicals were from Sigma-Aldrich (St. Louis, MO). Dispase II and collagenase I were obtained from Roche (Indianapolis, IN). Anti-BrdU antibody (GTX42641) was from GeneTex (Taipei, Taiwan). Anti-CD146 (ab75769), anti-Oct4 (ab18976), anti-nestin (ab6142), anti-CD31 (ab24590), anti-nucleostemin (ab70346), anti-collagen I (ab6308), and anti-collagen III antibody (ab6310) antibodies were from Abcam (Cambridge, MA). Phospho-Stat3 (Tyr705), STAT3, phospho-p44/42 MAPK (Erk1/2) (Thr202/Tyr204), and ERK antibody were purchased from Cell Signaling Technology (Danvers, MA). SB203580, PD98059, and STAT3 inhibitor (No. 573096) were purchased from Calbiochem (La Jolla, CA, USA).

### Animal studies

All animals were housed in an animal room under temperature control (24–25 °C) and a 12:12 light-dark cycle. Standard laboratory chow and tap water were available ad libitum. Experimental procedures were approved by the Mackay Memorial Hospital Review Board (New Taipei City, Taiwan) and were performed in compliance with national animal welfare regulations.

### Isolation and culture of TSPC

New Zealand white rabbits (6–8 months old, 3.0–4.0 kg) were used for the isolation of tendon cells. Achilles tendons were removed from the rabbits, by cutting through their bony attachments, and washed two times in sterile phosphate-buffered saline (PBS) containing 50 μg/ml gentamicin. The tendon and tendon sheath were cut into small pieces (1–2 mm^3^). Each 100 mg of tissue was then digested in a solution containing 3 mg/ml of type I collagenase and 4 mg/ml of dispase II in 1 ml balanced salt solution (BSS; Alcone) at 37 °C for 4 h. The digested tissues were washed three times in PBS, collected by centrifugation (800*g* for 10 min), placed into tissue culture dishes (Falcon Labware, NJ, USA) and resuspended in high-glucose DMEM, supplemented with 10% FBS and 50 μg/ml gentamycin, and maintained at 37 °C with 5% CO_2_. After 5 days, the medium was changed to remove the loosened tissue residues. For passaging, the tendon cells were harvested with 0.25% trypsin/EDTA and counted using a hemocytometer.

### Colony-forming efficiency

Approximately 2 × 10^3^ primary tendon cells were seeded into 25 T cell culture flasks (Corning), incubated with 10% FBS medium for 2 days, and their clonogenic capacity was determined in DMEM basal medium (2% FBS, 1% ITSE, 300 μg/ml l-glutamine, 1% antibiotic-antimicotic solutions), supplemented with 10 μM 29-mer, 34-mer, or peptide solvent (DMSO; dimethyl sulfoxide), for a further 10 days. The culture medium was changed every 3 days. Expression of TSPC markers by these expanded tendon cells was determined by immunostaining and western blot analysis.

### Immunocytochemistry

Cells cultured on slides were fixed with 4% paraformaldehyde, treated at 4 °C with methanol for 1 min, and then blocked with 1% goat serum and 5% BSA for 1 h. The cells were stained with antibodies to CD146 (1:50 dilution), nucleostemin (1:100 dilution), or BrdU (1:100 dilution) at room temperature (RT) for 3 h. The slides were subsequently incubated with FITC-donkey anti-rabbit IgG or Alexa Fluor® 647 Goat anti-mouse IgG (1:500 dilution; BioLegend, San Diego, CA) for 20 min and then counterstained with Hoechst 33258 for 6 min. The slides were rinsed with PBS with Triton X100 (0.5%) three times, mounted with FluorSave™ reagent (Calbiochem) and viewed with a Zeiss epifluorescence microscope.

### Biomechanical testing

After 3 weeks, the repaired Achilles tendon (*n* = 16) and the contralateral control tendons (*n* = 16) were harvested for mechanical evaluation. The specimens were dissected to remove the gastrocnemius/soleus muscle complex and soft tissues, leaving the intramuscular tendinous fibers, Achilles tendon, and calcaneal bone intact. The specimens were kept moist with normal saline during the entire testing procedure. The sagittal and transverse diameters of the mid-part of the callus were measured using a caliper to estimate the cross-sectional area [14]. Tendon fibers were fixed in a metal clamp by fine sandpaper. The calcaneal bone was fixed in a custom-made clamp at 30° dorsiflexion, relative to the direction of traction, as described previously [14, 15]. The mechanical testing machine (MTS Systems Corp., 14000 Technology Drive, Eden Prairie, MN) pulled the mounted tendon at a constant speed (0.1 mm/s) until failure, after a preload of 0.8 N had been applied. The data acquisition rate was 1/0.03 s. The ultimate tensile stress at tendon failure, expressed in newtons (N), was recorded during the process of failure testing. Young’s modulus (MPa) was calculated from the linear slope of the axial force–axial displacement curve.

### BrdU labeling in vitro

BrdU (final concentration, 10 μM) was added to the culture for 4 h. After fixing with 4% paraformaldehyde, the cells were exposed to cold methanol for 1 min and then treated with 1 N HCl at RT for 1 h before performing immunocytochemistry. The phenotype of TSPC was determined by immunostaining of nucleostemin.

### Western blot analysis

Cell lysis, SDS–PAGE, and antibodies used for immunoblotting were as described in a previous study [16]. The band intensity in immunoblots was evaluated with a Model GS-700 imaging densitometer (Bio-Rad Laboratories, Hercules, CA) and analyzed using Labworks 4.0 software.

### Quantitative real-time RT-PCR

The total RNA extraction, cDNA synthesis, and real-time PCR were performed as described previously [13]. Primers used in the experiment are listed in Table 1.

**Table 1:** Primers used in the real-time qPCR

Primers	Accession number	Sequences (5′-3′)
*Oct4* (*POU5F1*)	NM_001099957.1	F: TATGACTTCTGCGGAGCGAT
		R: TGCTCCAGCTTCTCCTTCTC
*nestin*	AB231855.1	F: GAGACCGAGTCTCAGGACAG
		R: CCCTCTCCAAGGGAACAGAG
*Col1a1*	XM_017348831.1	F: CTTCTGCGACATGGACACTG
		R: CACGTGCTTCTTCTCCTTGG
*Col3a1*	XM_002712333.3	F: GAAAGCCCTGAAGCTGATGG
		R: TGGGTAGTCTCACAGCCTTG
*Tenascin C*	FJ480400.1	F: CTGGGAACACGGTGGAGTAT
		R: TCGGTTGCAGTCAAGTCTCT
*Mkx*	XM_008268035.2	F: CCAGGACTGGAACCAGAGTT
		R: TGTGGAGGTTTGCGATAGGT
*Egr1*	XM_002710239.3	F: GTTTCACGTCTTGGTGCCTT
		R: TGAGCATGTCCCTCACAGTT

### Surgical procedure for rat Achilles tendon injury

To investigate the effects of the 29-mer peptide on tendon healing, a rat model of Achilles tendon injury was established, as reported previously [17]. Ten-week-old adult male Sprague-Dawley rats (initial body weight = 312 ± 11 g) were anesthetized by an intraperitoneal injection of xylazine (10 mg/kg). The left tendo-Achilles injury was created by full-thickness insertion of an 18-G needle through the tendo-Achilles, 1 cm proximal to the calcaneum attachment site. This created a horizontal wound which was flanked by intact tendon tissue to prevent the retraction of the severed ends. The skin incision was closed after the wound was irrigated with sterile saline. Treatments were applied to the area around the tendon lesion by subcutaneous injection with 150 μl of alginate gel mixed with 100 μM 29-mer or DMSO vehicle.

### Immunohistological staining

Formalin-fixed, paraffin-embedded tendon specimens were cut into 5-μm longitudinal sections, deparaffinized in xylene, and rehydrated in a graded series of ethanol concentrations. Slides were blocked with 10% goat serum for 60 min and then incubated with primary antibody against CD146 (1:50 dilution), nucleostemin (1:50 dilution), and CD31 (1:100 dilution) at room temperature (RT) for 2 h. The slides were subsequently incubated with the FITC-donkey anti-rabbit IgG and Alexa Fluor® 647 Goat anti-mouse IgG (1:500 dilution) for 20 min and then counterstained with Hoechst 33258 for 6 min and viewed with Zeiss epifluorescence microscope.

Deparaffinized tendon specimens were also blocked with 10% goat serum for 60 min and then incubated with antibody against CD146, collagen I, and collagen III (1:100 dilution, 37 °C for 3 h). The slides were subsequently incubated with the appropriate peroxidase-labeled goat immunoglobulin (1:500 dilution; Chemicon, Temecula, CA) for 20 min and then incubated with chromogen substrate (3,3′-diaminobenzidine) for 2 min before counterstaining with hematoxylin.

### In vivo detection of DNA synthesis

For the detection of cell expansion, BrdU was reconstituted in DMSO as stock (80 mM). One hundred fifty microliters of BrdU mixed with 350 μl of PBS was injected intraperitoneally into the rats at days 0, 3, and 5 after surgery. Tissue sections were treated with 1 N HCl at RT for 1 h, and DNA synthesis was assessed by anti-BrdU antibodies.

### Histological analysis

The tendon specimens were stained with hematoxylin and eosin (H&E). Photomicrographs of the tissue were captured through an Olympus IX71 light microscope and an Olympus XC10 camera (Japan). Four sections of each sample were selected and evaluated by two blinded observers to assess the tendon morphology according to a modified semi-quantitative grading score from 0 to 3. The score analyzed the fiber arrangement, fiber structure, nuclear roundness, cell density, infiltration of inflammatory cells and fibroblasts, and neovascularization [18–20]. According to this grading system, a perfectly normal tendon scored 0 and mild and moderate prevalence scored 1 and 2, whereas a score of 3 was assigned to a severely abnormal tendon.

### Statistics

The data were generated from three independent experiments. All numerical values are expressed as the mean ± SD. Comparisons of two groups were made using two-tailed Student’s *t* test. *P* < 0.05 was considered significant.

## Results

### The 29-mer stimulates the proliferation of CD146^+^ TSPC in culture

**Fig. 1:**
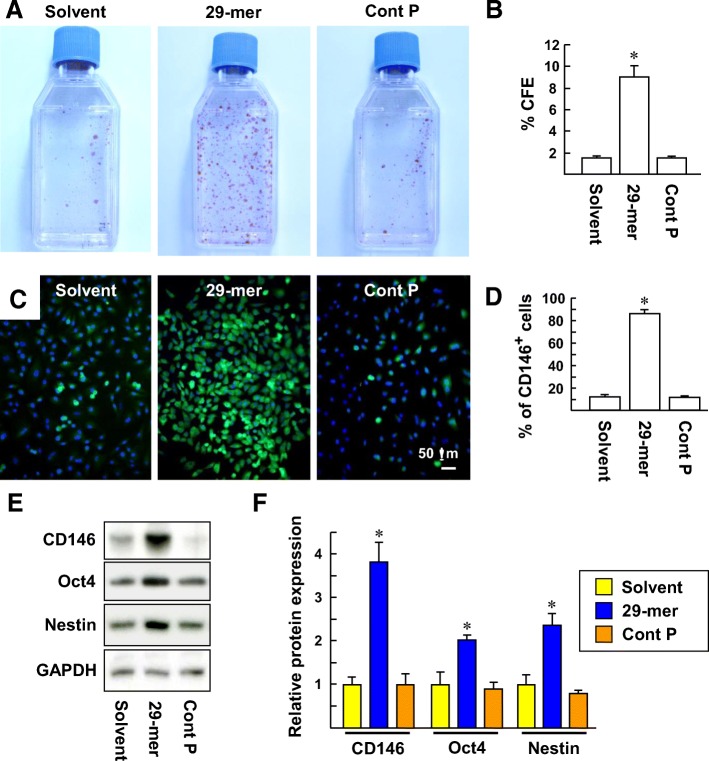
The 29-mer induces TSPC expansion among primary tendon cells. **a**, **b** Colony-forming efficiency (CFE). 2 × 10^3^ primary rabbit tendon cells were continuously cultivated in basal medium supplemented with 10 μM 29-mer, 34-mer (control peptide), or peptide solvent for 10 days. Representative images from three independent experiments. Three independent experiments were performed (*n* = 3, *P < .0005). **c**, **d** Immunofluorescence staining of colonies by CD146 antibody. Nuclei were stained by Hoechst 33258 (blue). The micrographs are representative of at least three independent experiments. The percentage of CD146^+^ cells per total cells in a colony was quantified. *P < 0.0001 versus solvent-treated cells. **e**, **f** Western blot analysis with antibodies as indicated. Representative immunoblots and densitometric analysis from three independent experiments are shown. The immunoblots were scanned and quantitated at individual sites and normalized to GAPDH. *P < 0.001 versus solvent-treated primary tendon cells

To verify the function of PSP, primary rabbit tendon cells were treated with the 29-mer, control peptide (34-mer), or peptide solvent (DMSO) for 10 days, as described in the “Materials” and “Methods” sections. Tendon cells stimulated with the 29-mer formed colonies (consisting of about 30–350 cells; Fig. 1a). Quantitatively, 29-mer-treated tendon cells had a greater clonogenic capacity than those of the solvent and control peptide-treated cells (Fig. 1b, 9.0 ± 1.1% versus 1.5 ± 0.2% and 1.5 ± 0.2%). CD146 is a TSPC marker and has been shown to be correlated with clonogenic capacity in vitro [3, 21]. Immunostaining of colonies by CD146 showed that most of the 29-mer-treated cells had a CD146-positive phenotype, approximately 7-fold more than the tendon cells treated by solvent or the control peptide (Fig. 1b, c). Western blot analysis showed that the levels of CD146 protein were 3.8-fold higher than the solvent control when the primary tendon cells were stimulated with the 29-mer for 10 days (Fig. 1d). Oct4 and nestin have been identified as TSPC markers [3, 21–23]. The 29-mer treatment also can result in greater amounts of Oct4 and nestin proteins than the solvent control (2-fold and 2.4-fold, respectively). Collectively, the 29-mer stimulates TSPC expansion in culture. The expression of TSPC markers is enriched by 29-mer treatment, implying that the expanded TSPC may still sustain their stemness.

### Sustained release of the 29-mer peptide promotes Achilles tendon healing

**Fig. 2:**
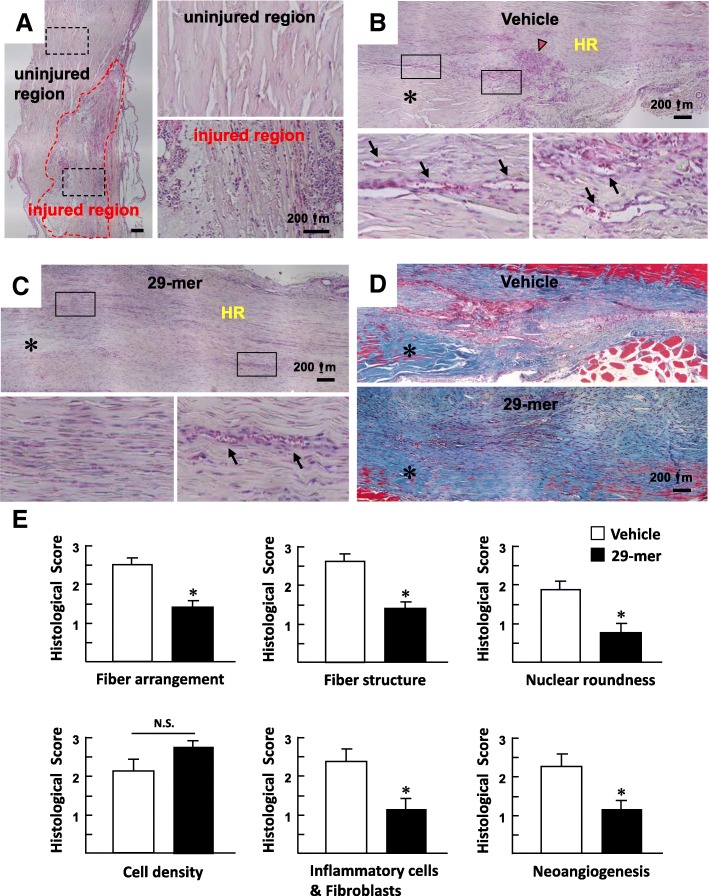
The 29-mer/alginate gel promotes Achilles tendon healing. **a** Representative H&E-stained longitudinal tendon sections 2 days after surgery. Rat Achilles tendon rupture was created by inserting an 18-G needle (the injured region is marked by a dashed line). The sections at higher magnification show normal tendon with a relative scarcity of cells among the collagen fibers. The injured region shows inflammation, marked by numerous blue-stained nuclei. **b**, **c** Representative H&E-stained sections 3 weeks after surgery. The 29-mer hydrogel treatment led to a dense, regular tendon tissue at the healing region (HR) compared to the disorganized tissue in tendons treated with vehicle hydrogel. Asterisk (*)—uninjured region. Arrowhead (red)—inflammatory matrix formed by high inflammatory cell invasion. Black arrow—neovessel. **d** Representative Masson’s trichrome-stained longitudinal tendon sections 3 weeks after surgery. The 29-mer-induced dense alignment of collagen fibers. **e** Histology scores of tendon healing 3 weeks after treatment. The histological scoring was performed double-blind. Fiber arrangement—scale of 0–3, 0 represents compacted and parallel, and 3 represents no identifiable pattern. Fiber structure—scale of 0–3, 0 represents continuous, long fibers, and 3 represents severely fragmented. Rounding of nuclei—scale of 0–3, 0 represents long spindle shape, and 3 represents severe rounding. Cell density—scale of 0–3, 0 represents normal pattern, and 3 represents severely increased. Fibroblasts/PMN/monocytes—1 (< 50 per field), 2 (51–100 per field), and 3 (101–150 per field). Vascularity—1 (< 5 per field), 2 (6–10 per field), and 3 (> 10 per field). High powered field (× 20). Results are expressed as mean ± SE of three independent experiments. *P < 0.001 versus vehicle-treated group. NS, not significant, *P* = 0.08

To investigate the therapeutic effect of the 29-mer on injured tendons, a rat model of Achilles tendon injury was established by full-thickness insertion of an 18-G needle horizontally through the tendo-Achilles [17]. On day 2 post-surgery, the needle insertion had led to inflammatory responses and necrotic tissue formation (Fig. 2a). Alginate gel loaded with the 29-mer was coated onto the tendon lesion to treat the tendon rupture. The hydrogel releases 90% of the loaded 29-mer in 5 days, as described in a previous report [12]. According to histological analysis of the H&E stained sections at 3 weeks postoperation, vehicle/alginate gel alone did not affect the self-healing, characterized by scar-like tissue, inflammatory matrix (marked by a red arrowhead), and numerous neovessels (marked by black arrows) in the healing region (HR; Fig. 2b). The 29-mer/alginate gel treatment improved wound-healing, with evidence of formation of densely aligned collagen fibers, less inflammatory cell invasion, and fewer neovessels in the HR (Fig. 2c). Masson’s trichrome staining confirmed that the 29-mer/alginate gel treatment led to dense and aligned collagen fibers parallel to the native tendon, in contrast to disorganized collagen formed in the vehicle/alginate group (Fig. 2d). Histological scoring analysis was performed by two blinded examiners. The average histology score for fiber arrangement, fiber structure, nuclear roundness, infiltration of inflammatory cells/fibroblasts, and neovascularization was significantly lower for the 29-mer/alginate gel group than the vehicle group 3 weeks after treatment (Fig. 2e; *P < 0.001). The higher cell density in the 29-mer/alginate gel group reflects accelerated TSPC proliferation. Collectively, the 29-mer displays efficacy in promoting tendon regeneration.

**Fig. 3:**
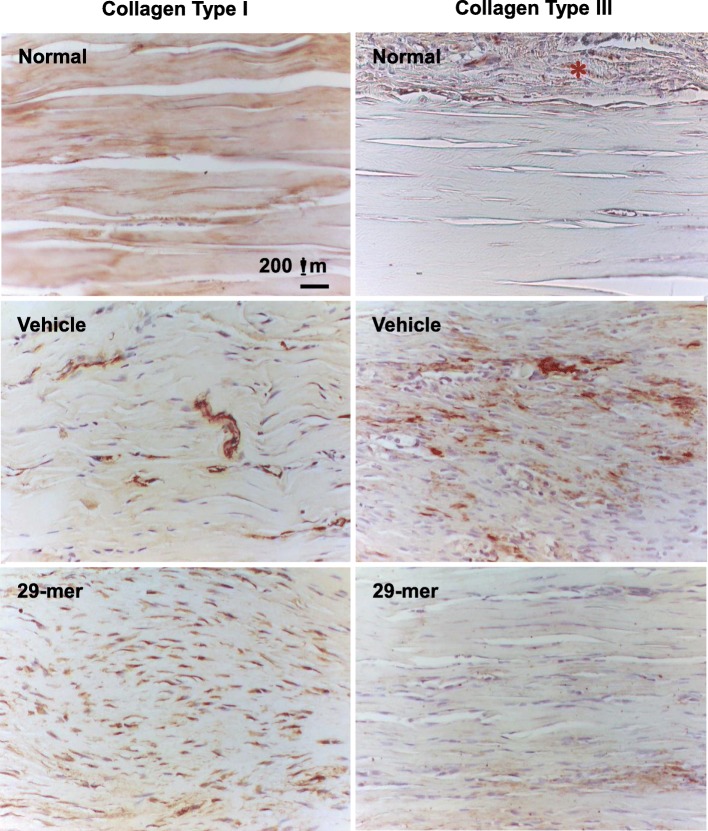
Immunohistochemical staining of type I and type III collagen after tendon healing for 3 weeks following vehicle or 29-mer/alginate gel treatment. Representative micrographs from two independent experiments (*n* = 6) are shown. Magnification, × 200. Note the strong expression of Col III in tendon peripheral tissue (marked with an asterisk)

Type I collagen (Col I) fiber bundles are major tendon matrix components and endow tendons with the ability to support tensile loading. Type III collagen (Col III) is typically over-expressed during the early inflammatory and proliferative stages of tendon healing. The increased content of Col III at later stages of tendon healing can cause thinner collagen fibers with a lower tensile strength [24]. IHC staining was used to compare the amounts of Col I and Col III at the tendon rupture site after healing for 3 weeks. As shown in Fig. 3, intact tendons featured predominantly Col I, with little Col III in the tendon matrix. The vehicle group displayed a weaker Col I deposition in the tendon matrix, but retained substantial Col III in the rupture site, reflecting the natural healing process. In contrast, the 29-mer/alginate gel treatment exhibited a strong expression of Col I in the tendon cells than the vehicle group. Col III expression in the 29-mer group was decreased to a level similar to intact tendons. The Col I/Col III staining and Masson’s trichrome staining (Fig. 2d) data imply that 29-mer treatment contributes to the tendon healing responses.

**Fig. 4:**
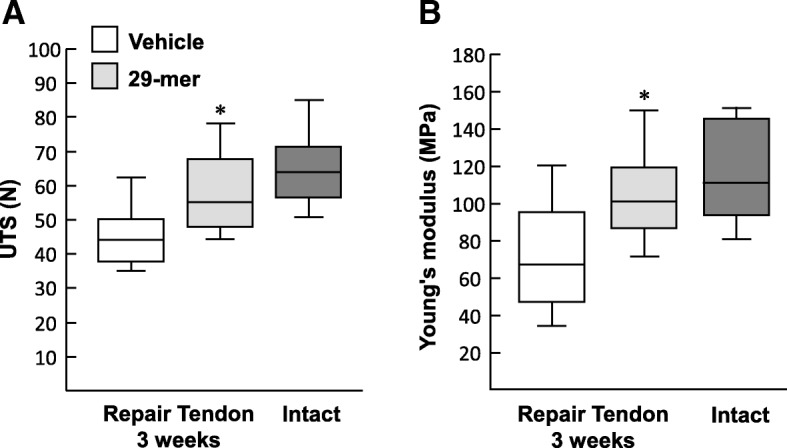
Biomechanical tests of normal tendon and regenerated tendons 3 weeks after surgery. Ultimate tensile stress (**a**) and Young’s modulus (**b**) of the 29-mer/alginate group (*n* = 8) and vehicle group (*n* = 8) were calculated from the axial force–axial displacement curve. The contralateral intact tendon was used as the control (*n* = 16). *P < 0.05 versus vehicle-treated group

We examined the biomechanical properties of repaired tendons to determine the efficacy of the 29-mer treatment. Sixteen rats underwent Achilles tendon surgery were randomized to either the 29-mer/alginate gel or vehicle treatment groups. Mechanical testing 3 weeks after the surgery showed that the ultimate tensile stress (UTS) and Young’s modulus of the repaired tendon in the 29-mer/alginate gel group reached 89.1% and 89.3%, respectively, compared to the contralateral intact tendons (Fig. 4). In contrast, the values of the UTS and Young’s modulus of the vehicle group were 68.7% and 61.1%, respectively, significantly lower than the 29-mer/alginate gel group (*P* < 0.05). In summary, the 29-mer/alginate gel treatment leads to increased tendon strength after healing for 3 weeks.

### Expanded TSPC induced by 29-mer retain the capacity for tenogenic differentiation

**Fig. 5:**
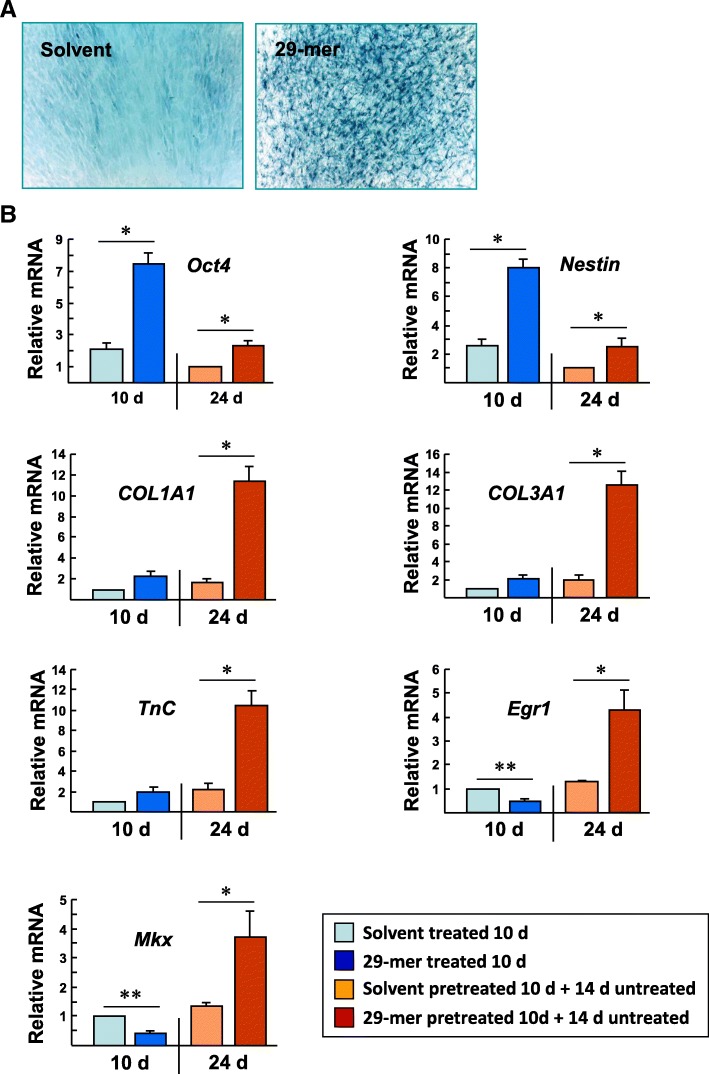
Tenogenic differentiation of TSPC expanded by the 29-mer. Primary isolated tendon cells were treated with the 29-mer or solvent for 10 days, and the cells were cultured in medium without the 29-mer or solvent for a further 14 days. **a** Representative Masson’s trichrome-stained micrographs of primary tendon cells pretreated with the 29-mer or solvent and stained on day 24. Data represent three independent experiments. **b** Real-time qPCR analysis of the levels of TSPC marker genes (*Oct4* and *nestin*) and teno-lineage marker genes (*Col1a1*, *Col3a1*, *Tnc*, *Egr1*, and *Mkx*) among primary tendon cells on days 10 and 24. *Gapdh* was used as a loading control. Data are representative of three independent experiments. *P < 0.001 versus solvent-treated cells. **P < 0.0001 versus solvent-treated cells

Next, we investigated whether the expanded TSPC, induced by the 29-mer, still preserve the capacity for tenogenic differentiation. Isolated primary tendon cells were treated with the 29-mer for 10 days to expand TSPC, followed by culture in basal medium without the 29-mer to induce tenogenic differentiation for a further 2 weeks. The 29-mer pretreatment increased collagen deposition, compared to solvent pretreatment (Fig. 5a; Masson’s trichrome staining). A real-time qPCR assay revealed that, during the 10 days of culture with the 29-mer, the levels of *Oct4* and *nestin* mRNA were 3.5- and 3.1-fold greater than in solvent-treated cells (Fig. 5b). Following the withdrawal of 29-mer for 14 days, the levels of *Oct4* and *nestin* mRNA declined substantially. Notably, the primary tendon cells pretreated by the 29-mer showed higher levels of expression of tenocyte lineage-related genes, including collagen type I (*Col1a1*), collagen type III (*Col3a1*), and tenascin-C (*Tnc*), than solvent-pretreated cells (Fig. 5b; approximately 4.6- to 6.5-fold; column 4 versus column 3). Two tendon lineage-related transcription factors, the zinc finger protein EGR1 (early growth response-1) and the Mohawk homeobox (Mkx) protein, have been shown to direct tendon differentiation and repair [25, 26]. Molecular analyses of ectopic *Egr1* or *Mkx* expression in murine C3H10T1/2 mesenchymal stem cells have shown that exposure to the 29-mer leads to increased expression of tendon lineage-related genes, including *Col1a1*, *Col3a1*, and *Tnc* [26, 27]. As shown in Fig. 5b, 29-mer treatment for 10 days led to 2.1-fold and 2.3-fold lower levels of *Egr1* and *Mkx* than the solvent controls, implying that the 29-mer prevents TSPC spontaneous tenogenic differentiation in vitro. However, inhibition of differentiation by the 29-mer is reversible when the peptide is withdrawn; a qPCR assay revealed significantly more *Egr1* and *Mkx* expression than in solvent-pretreated cells (3.4- and 2.7-fold greater; column 4 versus column 3). A similar trend was observed with significantly higher levels of expression of *Col1a1*, *Col3a1*, and *Tnc* in cells following 29-mer pretreatment. The data suggest that TSPC expanded by the 29-mer retain the capacity for tenogenic differentiation in vitro.

### The 29-mer promotes CD146^+^ TSPC proliferation in response to acute Achilles tendon injury

**Fig. 6:**
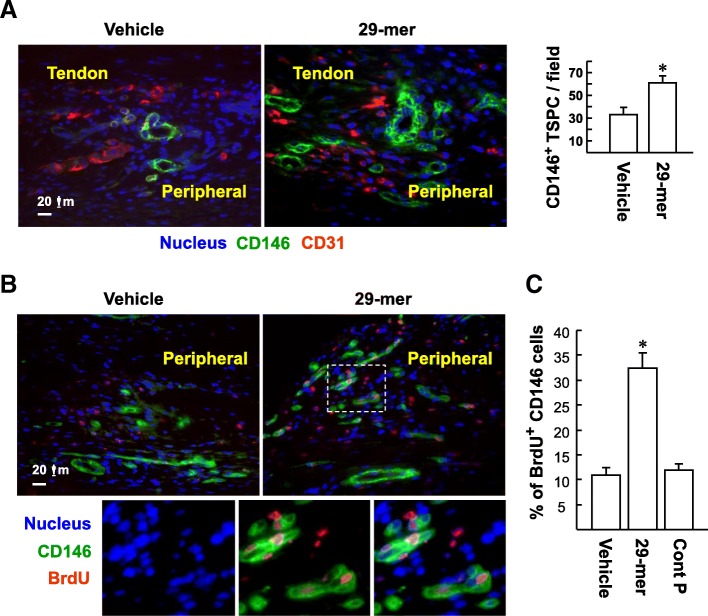
The 29-mer/alginate gel induces CD146^+^ TSPC proliferation in the peripheral region of the injured Achilles tendon. **a** Immunofluorescence analysis of the levels of CD146^+^ TSPC (green) and blood vessels (marked by CD31; red) at 2 days postoperation with the delivery of the 29-mer/alginate gel. Nuclei were visualized with Hoechst 33258 staining. Representative images are from six sections per rat tendon, with six rats per group. The digital image analysis of CD146^+^ TSPC was performed blinded on an average of six randomly selected × 400 magnification fields from each section, using a Zeiss epifluorescence microscope and Zeiss software. *P < 0.01 versus vehicle-treated group. **b** Double-immunofluorescence analysis of CD146 (green) and BrdU (red). Insets are the expanded TSPC detected by superimposing CD146, BrdU, and nuclear images using a digital program. Representative images are from three independent experiments with six rats per group. **c** Percentages of CD146/BrdU double-positive cells per total CD146^+^ cells. *P < 0.01 versus vehicle/alginate gel treatment

To seek evidence of the effect of the 29-mer on TSPC expansion in vivo, we monitored CD146^+^ TSPC expansion following tendon surgery combined with 29-mer/alginate gel delivery for 2 days. Immunofluorescence analysis revealed that the level of CD146^+^ TSPC was higher, at the peripheral region of the injured Achilles tendon, in the 29-mer/alginate gel group than the vehicle group (Fig. 6a; CD146^+^ TSPC per microscope field, 61.4 ± 6.0 versus 33.6 ± 7.3). In contrast, the levels of CD31^+^ vascular endothelial cells were not affected by the 29-mer or vehicle treatment (Fig. 6a). To investigate whether the accumulation of CD146^+^ TSPC is partly due to the mitogenic effect of the 29-mer, rats were injected intraperitoneally with BrdU to detect TSPC DNA synthesis immediately after tendon surgery. At day 2 after tendon wounding, immunofluorescence analysis confirmed that the 29-mer/alginate gel treatment resulted in higher levels of CD146/BrdU double-positive TSPC than the vehicle group (Fig. 6b, c; 32.4 ± 2.9% versus 10.9 ± 1.3%). The results imply that the 29-mer can impart mitogenic activity on CD146^+^ TSPC in injured Achilles tendons.

### The 29-mer causes accumulation of nucleostemin^+^ TSPC in the healing region of the Achilles tendon

**Fig. 7:**
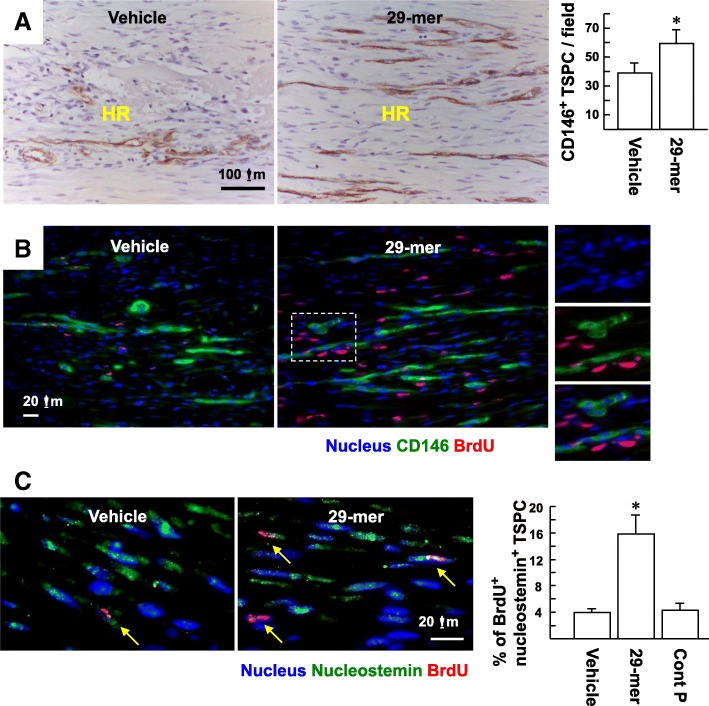
Immunohistochemical analysis of the distribution of various TSPC populations at 1 week postoperation. Tendons were treated with vehicle or 29-mer/alginate gel immediately after surgery. BrdU was injected intraperitoneally on days 0, 3, and 5. Tendons were harvested on day 7. **a** CD146 immunostaining (brown) and counterstaining with hematoxylin. Representative images are from three independent experiments. HR, healing region. The digital image analysis of CD146^+^ TSPC was performed blinded on an average of six randomly selected × 400 magnification fields from each section using a Nikon Eclipse 80i microscope equipped with a Leica DC 500 camera. *P < 0.05 versus vehicle-treated group. **b** CD146 and BrdU double immunostaining. Insets are the images before superimposition. Representative images are from three independent experiments. **c** Nucleostemin and BrdU double-immunostaining. Arrows indicate nucleostemin/BrdU double-positive TSPC. Representative images from three independent experiments with six rats per group are shown. Percentages of nucleostemin/BrdU double-positive cells per total nucleostemin^+^ cells were evaluated. *P < 0.002 versus vehicle group

One week after wounding, CD146 immunostaining of the Achilles tendons revealed large amounts of CD146^+^ TSPC in the HR of the 29-mer/alginate gel-treated tendons. In contrast, the amounts of CD146^+^ TSPC in the vehicle group were significantly lower (Fig. 7a; CD146^+^ TSPC per microscope field, 59.6 ± 9.2 versus 38.6 ± 6.9). The 29-mer effectively enhances CD146^+^ TSPC expansion into the HR, supporting rapid tendon wound healing. Interestingly, almost all of these CD146^+^ TSPC at the HR were stained negative for BrdU in the 29-mer and vehicle groups, even though there were large amounts of BrdU^+^ cells throughout the HR (Fig. 7b). It has been suggested that different TSPC populations exhibit different anatomical and temporal expression during rat Achilles tendon healing, such as nucleostemin^+^ TSPC throughout the HR at 1~2 weeks postoperatively [28]. Immunofluorescence staining indicated that almost all of the BrdU^+^ cells at the HR were stained positive for nucleostemin (Fig. 7c). More importantly, significantly more nucleostemin/BrdU double-positive cells were observed in the 29-mer/alginate gel-treated tendon than the vehicle control (15.8 ± 3.0% versus 4.0 ± 0.5%). Taken together, the data indicate that 29-mer not only stimulates the rapid expansion of CD146^+^ TSPC in both the tendon periphery and HR but also causes marked accumulation of nucleostemin^+^ TSPC at the HR to accelerate the wound-healing.

### The mitogenic effect of the 29-mer on nucleostemin^+^ TSPC is modulated by ERK2 and STAT3 signaling

**Fig. 8:**
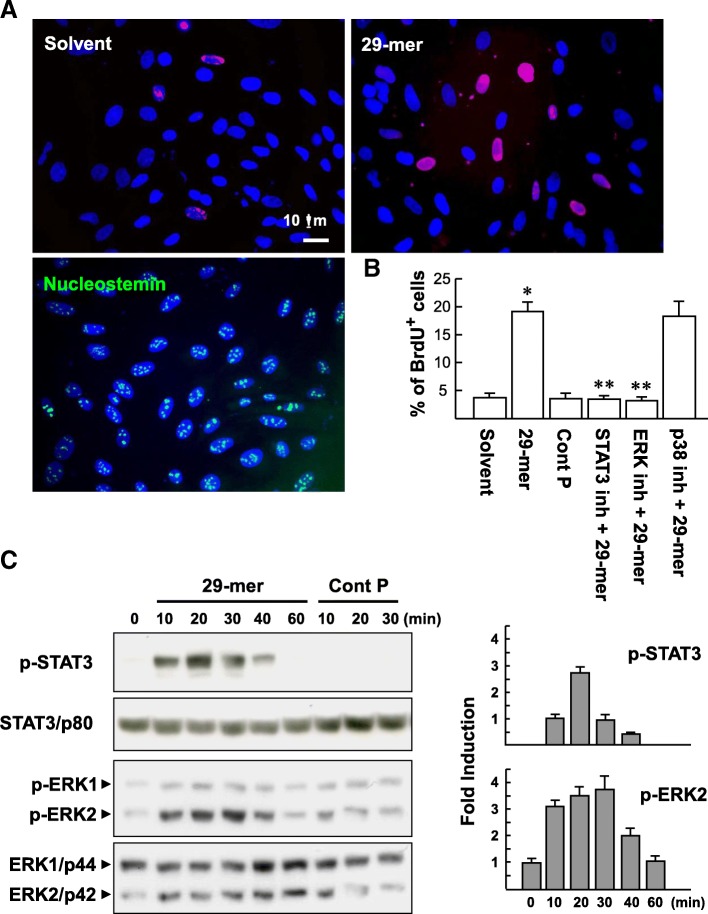
The 29-mer induces nucleostemin^+^ TSPC proliferation in an ERK- and STAT3-dependent manner. **a** BrdU-labeling assay. Primary rabbit tendon cells were cultured to near confluence in culture flasks for 14 days and then verified by nucleostemin immunostaining (> 98%). Cells were pretreated with PD98059 (10 μM; ERK inhibitor), 50 μM STAT3 inhibitor, or SB203580 (10 μM; p38 MAPK inhibitor) for 2 h before treatment with 10 μM 29-mer and 10 μM BrdU for another 4 h. BrdU (red)-labeled nuclei were detected by immunofluorescence microscopy. Representative images are from three independent experiments. **b** The percentages of BrdU^+^ cells per total cells (counterstained by Hoechst 33258; blue) were calculated from ten randomly selected microscopic fields in each treatment. Results are expressed as mean ± SD of three independent experiments. *P < 0.05 versus solvent-treated; **P < 0.05 versus 29-mer-treated cells. **c** Representative immunoblots and densitometric analysis of the effect of the 29-mer on phosphorylation of ERK2 and STAT3 in nucleostemin^+^ TSPC; the immunoblots were scanned and quantitated at individual sites and normalized to STAT3 and ERK2, respectively

Primary tendon cells were expanded for 14 days in culture and then exposed to the 29-mer (10 μM) for 24 h. TSPC purity was verified to be approximately 98% by nucleostemin staining (Fig. 8a). This also indicates that nucleostemin^+^ TSPC can be expanded as the major TSPC population from isolated primary rabbit tendon cells cultivated in basal culture medium. Cell proliferation was detected by BrdU pulse-labeling (4 h) and analyzed by BrdU immunostaining (red color). The assay revealed that stimulation by the 29-mer increased BrdU^+^ TSPC levels by 5.2-fold, compared with solvent treatment (Fig. 8b). Next, we used pharmacological inhibitors to explore the molecular basis of nucleostemin^+^ TSPC proliferation induced by the 29-mer. BrdU pulse-labeling assays revealed that pretreatment with STAT3 or ERK inhibitor suppressed the 29-mer-induced cell proliferation from 19.1 ± 1.7% to 3.4 ± 0.6% and 3.1 ± 0.6%, respectively (Fig. 8b). p38 MAPK inhibitor had no such effect. Meanwhile, the 29-mer can induce phosphorylation of ERK2 and STAT3 in nucleostemin^+^ TSPC. The immunoblots revealed that phosphorylation of STAT3 and ERK2 caused by the 29-mer occurred 10~40 min after the treatment (Fig. 8c). However, the 34-mer control peptide did not show these effects. Collectively, the findings imply that the 29-mer induces nucleostemin^+^ TSPC expansion in vitro via activation of the ERK and STAT3 signaling pathways.

## Discussion

Insufficient numbers of resident TSPC are activated in response to an acute tendon injury, and this may limit the healing process. This study is the first to show that the PSP 29-mer can promote the clonogenicity of CD146^+^ TSPC and proliferation of nucleostemin^+^ TSPC among cells isolated from the rabbit Achilles tendon. Moreover, we provide evidence that a single injection of the 29-mer hydrogel into a damaged tendon can induce expansion of CD146^+^ TSPC and nucleostemin^+^ TSPC at the early regenerative stage (2~7 days postoperative). Our study has demonstrated, in vitro and in vivo, that the 29-mer is a newly identified mitogen for TSPC.

In this study, an injectable alginate gel was used to provide sustained release of the 29-mer in the damaged tendon, leading to improved tendon repair. Alginate gel as a vehicle displays biocompatible, nontoxic, and biodegradable properties [29]. Our previous study showed that a single injection of the 29-mer/alginate gel can stimulate muscle satellite cell proliferation significantly to promote rat soleus muscle regeneration [12]. Despite these advantages, this previous experiment revealed that ~ 90% of the 29-mer was released from the alginate gel within 5 days in vitro [12]. Nonetheless, herein, our day 2 and day 7 histological data indicate that the sustained release of 29-mer for a few days was sufficient to augment CD146^+^ TSPC and nucleostemin^+^ TSPC in the niche and HR. The results support the hypothesis that the growth factor stimulates a sufficient level of CD146^+^ TSPC at the early phase of tendon wound healing, playing a crucial role in relieving disorganized collagen formation in the HR [3].

Healing of the tendons begins with the inflammatory phase that shows accumulation of hemorrhages and leukocytes and local synthesis of chemotactic and angiogenic factors [30]. The reparative phase begins later, characterized by the initiation of TSPC proliferation and migration of TSPC/tenocytes into the wound [30, 31]. Due to the limited self-healing capacity of the tendons, the healed tendon shows disorganized fiber architecture and scar tissue with disrupted collagens [3, 30]. These result in reduced elasticity, reduced mobility, and an increased propensity for the recurrence of injury [32]. In this study, the 29-mer hydrogel treatment accelerated TSPC proliferation that initially overlapped the inflammatory phase (day 2 to day 7), thereby leading to a decrease in fibrosis and scar tissue formation. The incomplete description of the role of 29-mer in the inflammatory phase of tendon healing is a limitation of this study. In this regard, PEDF reportedly can reduce inflammation in vitro and in vivo. For example, PEDF induces expression of the anti-inflammatory cytokine interleukin 10 (IL-10) by human macrophages [33]. PEDF treatment can reduce retinal inflammation in a rat model of diabetes [34]. Interestingly, our previous animal study found that PSP 44-mer (a sequence containing the 29-mer) can reduce inflammatory responses in acute liver injury through its protective effect on hepatocytes [13]. Whether the 29-mer is able to regulate the properties of inflammatory cells during tendon injury warrants additional research.

It has been reported that the nucleostemin is a TSPC marker because its expression is eliminated in tenocytes [35]. In this study, the 29-mer increased the number of nucleostemin^+^ TSPC that were observed on day 7 post-injury. Nucleostemin^+^ TSPC have been shown to retain a high self-renewal capacity in vitro and express tenocyte-related markers, such as collagen I, collagen III, and Tnc [36]. Because collagen I and collagen III are important structural protein in tendons, the nucleostemin^+^ TSPC have a direct impact on tendon repair.

The relationship between CD146^+^ TSPC and nucleostemin^+^ TSPC remains elusive. There is still no evidence that CD146^+^ TSPC can differentiate to nucleostemin^+^ TSPC. A study discovered that stem cells derived from the human anterior cruciate ligament (hACL) and medial collateral ligament (hMCL) express nucleostemin, but not CD146 [37]. Interestingly, a recent report indicates that rat tendon injury leads to a large number of nucleostemin^+^ TSPC throughout the HR at 1 and 2 weeks post-injury [28]. The different TSPC populations have been suggested to be involved in a time-controlled tendon repair process [3, 28].

The present cell signaling study exploits nucleostemin^+^ TSPC rather than CD146^+^ TSPC. CD146^+^ TSPC are rare (~ 0.8%) in the rat patellar tendon [3]. Our histological sections also revealed that CD146^+^ TSPC numbers were extremely low in the uninjured rabbit Achilles tendon, and there was a rapid loss of stemness during culture in basal medium. PSP, as full-length PEDF, has been shown to initiate signaling by binding to the cell surface receptor, patatin-like phospholipase domain-containing protein 2 (PNPLA2). PNPLA2 receptor is essential for PEDF/PSP to induce mitogenic signaling on human embryonic stem cells and neural stem cells, as well as antiapoptotic signaling on hepatocytes [13, 38, 39]. Further studies are warranted to determine the expression of PNPLA2 in TSPC and the involvement of PNPLA2 in mediating PEDF/29-mer mitogenic signaling. Our study found that STAT3 signaling was critical for the induction of nucleostemin^+^ TSPC proliferation by the 29-mer. Our previous findings show that PSP enhances the proliferation of limbal stem cells and satellite cells by activating STAT3 signaling [11, 12]. In addition, phosphorylation of STAT3 has been found to be crucial for the proliferation of myoblasts and satellite cells induced by bFGF, leukemia inhibitory factor (LIF), and IL-6 [40, 41]. STAT3 is a transcription factor that regulates several targets closely associated with cell cycle progression, including cyclin D1 and SOCS3 [12, 42]. To our knowledge, there has been no report addressing the role of STAT3 signaling on TSPC proliferation. Our finding also implies that ERK2 activation is crucial for TSPC proliferation in vitro. This multiple signaling is reminiscent of the proliferative responses induced in satellite cells by the 29-mer [12]. ERK signaling as STAT3 is also involved in cyclin D1 expression by satellite cells [12].

## Conclusions

Growth factors accelerate TSPC expansion in the early phase of tendon wound-healing as a critical mechanism for improving tendon repair. This study shows that the PSP 29-mer displays mitogenic activity and regulates the resident TSPC, in response to acute tendon rupture, and facilitates tendon recovery of a higher quality in the animal model. The 29-mer may be a novel therapeutic remedy for acute tendon injury.

## Abbreviations

ASCs: Adipose-derived mesenchymal stem cells
BM-MSCs: Bone marrow mesenchymal stromal cells
BrdU: 5-Bromo-2′-deoxyuridine
BSS: Balance salt solution
*Col1a1*: Collagen type I
*Col3a1*: Collagen type III
CTGF: Connective tissue growth factor
DMEM: Dulbecco’s modified Eagle’s medium
DMSO: Dimethyl sulfoxide
EGR1: Early growth response-1
ERK: Extracellular signal-regulated kinase
FBS: Fetal bovine serum
FGF: Fibroblast growth factor
hACL: Human anterior cruciate ligament
hMCL: Medial collateral ligament
HR: Healing region
IGF: Insulin-like growth factor
IL-10: Interleukin 10
ITSE: Insulin–transferrin–sodium selenite
LIF: Leukemia inhibitory factor
MAPK: Mitogen-activated protein kinase
*Mkx*: Mohawk homeobox
Oct4: Octamer-binding transcription factor 4
PBS: Phosphate-buffered saline
PCR: Polymerase chain reaction
PDGF: Platelet-derived growth factor
PEDF: Pigment epithelial-derived factor
PNPLA2: Patatin-like phospholipase domain-containing protein 2
PRP: Platelet-rich plasma
PSP: Pigment epithelial-derived factor-derived short peptide
RT: Room temperature
Scx: Scleraxis
Sox9: SRY-box containing gene 9
STAT3: Signal transducer and activator of transcription 3
*Tnc*: Tenascin-C
TSPC: Tendon stem/progenitor cells
UTS: Ultimate tensile stress
